# Conversion Surgery in Metastatic Gastric Cancer and Cancer Dormancy as a Prognostic Biomarker

**DOI:** 10.3390/cancers12010086

**Published:** 2019-12-30

**Authors:** Hun Jee Choe, Jin Won Kim, Song-Hee Han, Ju Hyun Lee, Sang-Hoon Ahn, Do Joong Park, Ji-Won Kim, Yu Jung Kim, Hye Seung Lee, Jee Hyun Kim, Hyung-Ho Kim, Keun-Wook Lee

**Affiliations:** 1 Department of Internal Medicine, Seoul National University Bundang Hospital, Seoul National University College of Medicine, Seongnam 13620, Korea; 2 Department of Pathology, Seoul National University Bundang Hospital, Seoul National University College of Medicine, Seongnam 13620, Korea; 3 Department of Pathology, Dong-A University Hospital, Dong-A University College of Medicine, Busan 49201, Korea; 4 Department of Surgery, Seoul National University Bundang Hospital, Seoul National University College of Medicine, Seongnam 13620, Korea; 5 Department of Surgery, Seoul National University Hospital, Seoul National University College of Medicine, Seoul 03080, Korea

**Keywords:** gastric cancer, conversion surgery, cancer dormancy, nuclear receptor NR2F1

## Abstract

The role of conversion surgery in metastatic gastric cancer remains unclear. Cancer dormancy markers might have a role in predicting the survival in patients with conversion surgery. We identified 26 patients who went through conversion surgery, i.e., a curative-intent gastrectomy with metastasectomy after chemotherapy in initially metastatic gastric cancer. As controls, 114 potential candidates for conversion surgery who only received chemotherapy were included for the propensity score matching. Conversion surgery showed a significantly longer overall survival (OS) compared with only palliative chemotherapy (median—43.6 vs. 14.0 months, respectively, *p* < 0.001). This better survival in the conversion surgery group persisted even after propensity matching (*p* < 0.001), and also when compared to patients with tumor response over 5.1 months in the chemotherapy only group (*p* = 0.005). In the conversion surgery group, OS was longer in patients with R0 resection (22/26, 84.6%) than without R0 resection (4/26, 15.4%) (median—not reached vs 22.1 months, respectively, *p* = 0.005). Although it should be interpreted with caution due to the primitive analysis in a small population, the positive expression of NR2F1 showed a longer duration of disease-free survival (DFS) after conversion surgery (*p* = 0.016). In conclusion, conversion surgery showed a durable OS even in patients with initially metastatic gastric cancer when R0 resection was achieved after chemotherapy.

## 1. Introduction

The incidence of gastric cancer is widely varied geographically. Despite the trend of steady decline and country-specific disparity, it remains to be the fifth most prevalent cancer and the third cause of death worldwide [1,2]. Curative operation is the treatment of choice for resectable diseases. As for metastatic gastric cancer, systemic chemotherapy is the standard treatment modality. However, the prognosis remains poor with a median survival of about 12 months, despite recent advancements made in chemotherapeutics, including molecular targeting agents and cancer immunotherapy [3].

Conversion surgery is a term for operative resection of primary or metastatic lesions with a curative intent, after confirming either a complete response (CR) or partial response (PR), following several cycles of palliative chemotherapy. Although addition of gastrectomy (without metastasectomy) to palliative chemotherapy in metastatic gastric cancer did not show survival benefit when compared with chemotherapy only in the previous REGATTA trial [4], there have been recent attempts to conduct surgery in selected patients with a good initial response to palliative chemotherapy [5,6,7,8,9,10,11,12]. It has been shown to improve the prognosis in a few retrospective studies. However, whether this improvement of survival is attributed to conversion surgery or good tumor biology in patients undergoing conversion surgery remains unclear. The characteristics of patients that might benefit from conversion surgery also remain unknown.

Cancer dormancy is a clinical phenomenon in which the metastatic disease develops years or even decades after successful treatment with curative surgery and adjuvant treatment [13]. Dormant tumor cells might stay in the quiescent state for many years as solitary tumor cells or micrometastases that are not clinically apparent. Cancer dormancy could be associated with early or late recurrence after conversion surgery in patients with initial systemic metastasis.

In this study, we compared the outcomes of patients with metastatic gastric cancer who subsequently underwent conversion surgery after palliative chemotherapy, with patients who only received palliative chemotherapy using propensity score analysis. We also investigated whether the expression of cancer dormancy markers might play a role in predicting survival in patients receiving conversion surgery as a pilot study.

## 2. Results

### 2.1. Patients’ Demographic Data

Patient characteristics and the clinicopathologic findings are shown in Table 1. In the conversion surgery group, 6 patients (23.1%) were ≥70 years old, and 10 patients (38.5%) had macroscopic peritoneal dissemination. Of these, the favorable response of CR or PR to palliative chemotherapy was seen in 17 patients (65.4%). In the chemotherapy only (the control) group, 31 patients (27.2%) were ≥70 years old. A slightly higher portion of patients had macroscopic peritoneal dissemination (50.9%), as compared to the conversion surgery group (38.5%). CR or PR was achieved in the lower proportion of patients with palliative chemotherapy (51.8%). Both groups were balanced after propensity score matching (1:2) in terms of baseline characteristics. 

All patients received a fluoropyrimidine in combination with a platinum analogue as a first-line palliative chemotherapy in both groups. Those who showed an overexpression of HER2 also received trastuzumab. All conversion surgeries were conducted during the first-line chemotherapy, except in one patient who received conversion surgery during the second-line chemotherapy. The median duration of first-line chemotherapy before conversion surgery was 5.1 months. After conversion surgery, the first-line chemotherapy was continued in 13 patients (50%) as a maintenance therapy.

In the conversion surgery group, R0 resection was achieved in 22 patients (84.6%). Pathologic CR was shown in 2 patients (7.7%). Subtotal gastrectomy, total gastrectomy, and extended total gastrectomy were performed in 42.3%, 30.8% and 26.9%, respectively. Lymphatic invasion, vascular invasion, and perineural invasion were present in 80.8%, 42.3% and 65.4%, respectively (Table 2). At the first diagnosis before palliative chemotherapy, category 2 was the most prevalent biological disease status (42.3%), followed by category 4 (23.1%), category 1 (19.2%), and category 3 (15.4%). At the time of conversion surgery, CR, PR, stable disease (SD), and not evaluable (NE) had been established with chemotherapy in 2 (7.7%), 15 (57.7%), 3 (11.5%) and 6 (23.1%) patients, respectively.

### 2.2. Survival Outcomes of Conversion Surgery and Palliative Chemotherapy Only Group

**Figure 1:**
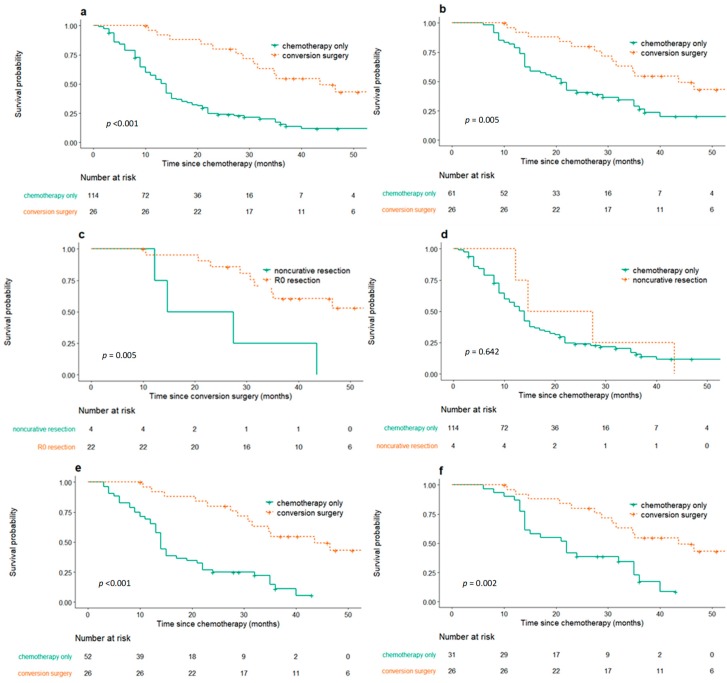
Kaplan–Meier curve for overall survival (OS) in patients who received conversion surgery and only chemotherapy. (**a**) Comparison of OS after chemotherapy in patients who received conversion surgery (*n* = 26) and those that only received chemotherapy (*n* = 114) (*p* < 0.001). (**b**) Comparison of OS after chemotherapy in patients who received conversion surgery (*n* = 26) vs. subgroup of patients in the chemotherapy only group whose tumor responded to or were stabilized with chemotherapy for 5.1 or more months (*n* = 61) (*p* = 0.005). (**c**) Comparison of OS in patients who received R0 resection (*n* = 22) vs. noncurative resection in conversion surgery (*n* = 4) (*p* = 0.005). (**d**) Comparison of OS after chemotherapy in patients who received noncurative resection in conversion surgery (*n* = 4) vs. only chemotherapy (*n* = 114) (*p* = 0.642). (**e**) After propensity score matching, comparison of OS after chemotherapy in patients who received conversion surgery (*n* = 26) vs. only chemotherapy (*n* = 52) after propensity score matching (*p* < 0.001). (**f**) After propensity score matching, comparison of OS after chemotherapy in patients who received conversion surgery (*n* = 26) vs. subgroup of patients with tumor response of 5.1 or more months after propensity score matching (*n* = 31) (*p* = 0.002).

The median follow-up duration was 36.1 months in the conversion surgery group and 13.0 months in the palliative chemotherapy only group. Overall survival (OS) was significantly longer for patients who received conversion surgery compared to those who only received palliative chemotherapy (Median OS: 43.6 months, 95% confidence interval [CI], 31.6–not reached [NR]; 14.0 months, 95% CI 11.0–15.0, respectively, *p* < 0.001, Figure 1a). The median duration of palliative chemotherapy before conversion surgery was 5.1 months, whereas 53 of 114 patients (46.5%) who only received chemotherapy had tumor progression before 5.1 months, while on chemotherapy. Thus, to avoid a potential selection bias, additional comparison was made between all patients in the conversion surgery group and the subgroup of patients in the chemotherapy only group whose tumor responded to or were stabilized with chemotherapy for greater than 5.1 months. This latter group of patients demonstrated a median OS of 21.0 months (95% CI 15.0–32.0), which nevertheless was appreciably shorter than that of the conversion surgery group (*p* = 0.005, Figure 1b).

Out of 26 patients that underwent conversion surgery, 22 patients received R0 resection. Cancer recurred in 15 of 22 patients (68.2%) that underwent conversion surgery with R0 resection. In those who underwent successful R0 resection, disease-free survival (DFS) from conversion surgery was 15.1 months (95% CI 7.5–NR). OS was longer in the R0 resection group (median value—NR, 95% CI 34.8–NR) compared with those without successful resection (21.1 months, 95% CI 12.2–NR, *p* = 0.005, Figure 1c, Table S1). There was no statistical difference in OS from the initial palliative chemotherapy between the group who received noncurative resection (21.1 months, 95% CI 12.2–NR) and the group who only received chemotherapy (14.0 months, 95% CI 11.0–15.0) (*p* = 0.642, Figure 1d).

Patients who received R0 resection in conversion surgery were further analyzed according to the initial biological disease status before palliative chemotherapy, chemotherapy duration before conversion surgery, tumor response to chemotherapy at conversion surgery, and postoperative pathological staging. A shorter duration of chemotherapy before conversion surgery was associated with a longer duration of DFS (*p* < 0.001) from conversion surgery. A less advanced pathological stage was also associated with DFS from conversion surgery (*p* = 0.005). In contrast, the between-group differences in DFS regarding the initial biological disease status before palliative chemotherapy were not clinically significant (*p* = 0.071). Tumor response to chemotherapy was not associated with DFS from conversion surgery in the conversion surgery group (*p* = 0.712), but was a prognostic factor in the chemotherapy only group (OS and progression-free survival (PFS): *p* = 0.005, *p* = 0.003, respectively)**.** The characteristics and clinical course of patients who showed long-term survival (>3 years) among patients with conversion surgery are described in Table 3.

### 2.3. Propensity Score Matching Analysis

Propensity score matching analysis was conducted with the purpose of balancing any confounding covariates, including initial biological disease status category before palliative chemotherapy, best response to chemotherapy, age, and sex. OS in patients who received conversion surgery and those that only received chemotherapy was 43.6 months (95% CI 31.6–NR) and 14.0 months (95% CI 13.0–21.0), respectively, after propensity score matching (*p* < 0.001, Figure 1e). Subgroup analysis was performed with the selected group of patients in which continuous tumor response to chemotherapy was obtained for 5.1 months or longer in the matched cohort, to reduce selection bias. The median OS was 22 months in this group (95% CI 14.0–35.0), which was also shorter than that in the conversion surgery group (*p* = 0.002, Figure 1f).

### 2.4. Mortality and Morbidity of Conversion Surgery

Of the 26 patients who received conversion surgery, there was no treatment-related mortality. Postoperative morbidity was evaluated according to the revised Clavien–Dindo classification of surgical complications [14]. Overall, 19.2% of patients had an adverse event with grade II or III; one pleural effusion (grade IIIa), one serous leakage (grade II), one pulmonary thromboembolism, and deep vein thrombosis (grade II), one ileus (grade II), and one pneumonia (grade II).

### 2.5. Cancer Dormancy Marker Expression

For the group of patients that went through conversion surgery, tissue microarray (TMA) was performed for the expression of cancer dormancy markers in 18 out of 26 initial biopsy specimens. Although positive expression (moderate to strong) of NR2F1 was identified in only 4 patients, the expression of NR2F1 was correlated with DFS after conversion surgery (*p* = 0.016). No significant differences in DFS were observed as per the expression of NANOG and MIG6 (*p* = 0.909, *p* = 0.314, respectively, Figure S1).

## 3. Discussion

The prognosis for those undergoing systemic therapies only for gastric cancers who were either initially diagnosed as metastatic or having developed recurrence after initial curative resection was dismal, in spite of recent advancements made in targeted and immune-based therapies [3]. Accordingly, there have been attempts to proceed to conversion surgery in metastatic gastric cancer in an effort to add survival benefit to chemotherapy even when systemic treatment only could at least temporarily control the microscopic disease [4,5,6,7,8,9,10,11,12].

The strengths of a well-designed randomized trial are indisputable; however, in studies involving surgical cases, such a study is difficult [15]. It is particularly difficult to undertake a randomized controlled study design when involving surgical cases of advanced gastric cancer. This is because surgery is currently the only option for curative treatment in advanced gastric cancer. Consequently, previous reports that studied conversion surgery in metastatic gastric cancer have adopted observational study models [4,6,7,8,10,15,16,17,18,19,20]. For this reason, most previous studies have obvious inherent limitations associated with selection bias in patients who received conversion surgery, i.e., better initial response to systemic chemotherapy or a lesser degree of peritoneal seeding, compared with the group who only received palliative chemotherapy. Furthermore, due to the inadequate tumor sample data in previous retrospective studies, there had not been attempts to define specific biological subgroups of patients who might benefit from conversion surgery.

In this study, we have implemented a propensity score modeling, producing a matched cohort based on baseline characteristics, as well as the extent of initial biological disease, before palliative chemotherapy and response to chemotherapy. Our findings were consistent with previous studies that advocated conversion surgery if R0 resection could be achieved, even after adjusting for potential confounding factors [9,10,12,21,22]. It is noteworthy that patients who underwent conversion surgery exhibited a longer survival rate after additionally excluding early disease progression in the chemotherapy only group. In addition, a shorter duration of preoperative chemotherapy was an independent predictor of DFS from conversion surgery, which was contradictory to the previous study [21]. This might in part be attributable to better chemotherapy efficacy in the subgroup of patients with a shorter duration of preoperative chemotherapy, leading to tumor shrinkage that facilitated conversion surgery [23]. Another explanation can be lead-time bias, which indicates that a shorter duration of chemotherapy before conversion surgery leads to a longer DFS after conversion surgery. Although there was no significant interaction between the initial biological disease status category before palliative chemotherapy and OS in the conversion surgery group, OS was significantly longer in patients who had reached less advanced pathological staging, emphasizing the role of systemic chemotherapy in clinical efficacy for tumor control and down-staging of the tumor for the improvement of the R0 resection rate.

In the initial phases of the metastatic disease, which is a candidate of conversion surgery but is very likely to have microscopic metastasis, cancer dormancy could be associated with recurrence after macroscopic curative resection. NR2F1 is an example of a cancer dormancy marker. It was an orphan nuclear receptor that was thought to be linked to longer DFS in breast and prostate cancer, undergoing a prolonged asymptomatic dormancy status before resuming metastatic growth [24,25]. Based on our results, a positive expression of NR2F1 in the initial biopsy specimen conferred a survival benefit in patients that went through conversion surgery, although this analysis was too primitive to provide any evidence, due to the small sample size. The small sample size also limited our ability to demonstrate the statistical significance as prognostic predictors in NANOG, MIG6, and PERK.

The present study has some limitations. First, this study might have some inherent biases due to the retrospective nature. Nevertheless, we tried to compare patients who received conversion surgery objectively by propensity score matching and excluding early disease progression in the control group. Some of the subgroup analyses were novel findings in our study. Second, preoperative staging without staging laparoscopy could have resulted in over-staging, particularly in cases of peritoneal seeding. Diagnostic staging laparoscopy and re-evaluation of peritoneal metastasis with staging laparoscopy might be helpful in improving the diagnostic accuracy and optimizing candidates for conversion surgery [26,27]. Third, the clinical efficacy of cancer dormancy marker expression in conversion surgery cannot be definitively concluded, given the relatively small sample size of this study. Even though the expression of NR2F1 was statistically significant as a good prognostic marker, there were only four patients who expressed NR2F1 among those who received R0 resection in the conversion surgery group. Other cancer dormancy markers that might have also played a role in determining the prognosis had limited power to show a statistically significant difference due to the small number of patients included. The number of patients in the conversion surgery cohort itself, however, was not small as compared to previous studies. Therefore, adopting cancer dormancy markers as a prognostic marker in conversion surgery remains a promising option. Fourth, cancer dormancy markers were evaluated only in the conversion surgery group and those who had only received chemotherapy did not have comparable tumor tissues for tumor dormancy marker staining. In the future, studies with a larger sample size comparing the various aspects of cancer dormancy marker expression in both the conversion surgery group and chemotherapy only group might be warranted for developing a clearer criterion in selecting patients apt for conversion surgery with better outcomes. Nonetheless, to the best of our knowledge, this is the first study to adopt the cancer dormancy concept in patients undergoing gastric conversion surgery, to date.

## 4. Patients and Methods

### 4.1. Patients with Conversion Surgery

**Figure 2:**
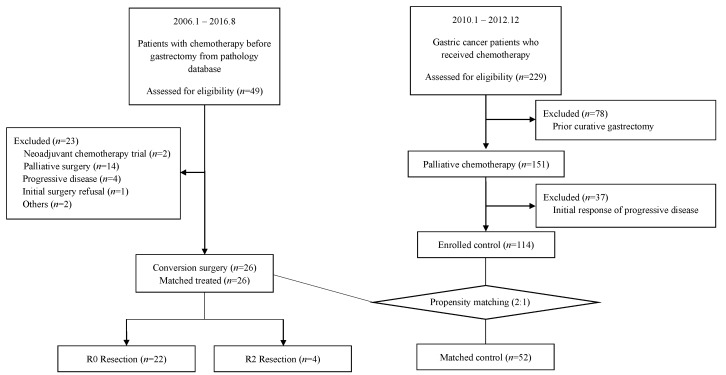
Consort diagram of the study.

Forty-nine patients with initially metastatic gastric cancer who underwent gastrectomy after palliative chemotherapy between January 2006 and August 2016 at Seoul National University Bundang Hospital (SNUBH) were identified from the pathology database. Patients were eligible for inclusion if they had a histological confirmation of adenocarcinoma in the initial biopsy, which was initially metastatic and had curative-intent gastrectomy or metastasectomy, after two or more cycles of chemotherapy. The exclusion criteria were the history of neoadjuvant chemotherapy in initially resectable disease and surgery with palliative intent, i.e., bleeding control. The choice of palliative chemotherapy was based on physicians’ preference. Twenty-six patients were finally included in the conversion surgery group (Figure 2).

### 4.2. Control Patients for Propensity Score Analysis

As for the control group in the propensity score analysis, 229 patients who received palliative first-line chemotherapy, from January 2010 to December 2012 at SNUBH, were initially screened. Of these, 78 patients who showed a metastatic recurrence after previous curative gastrectomy were excluded. Thirty-seven patients with the disease progression at first response evaluation as a result of primary resistance to palliative chemotherapy were also excluded (Figure 2). After excluding these 115 ineligible patients, 114 patients were considered as the potential candidates for conversion surgery. To mitigate selection bias for conversion surgery and the potential confounding factors, we adjusted for the baseline characteristics of the selected patients by propensity score analysis. Propensity scores were generated with the dependent variables “initial biological disease category before palliative chemotherapy” and “best responses to chemotherapy”, which were clinically relevant covariates prior to conversion surgery. Other matched variables included “age” and “sex”. Patients were then 1:2 matched without replacement into conversion surgery and palliative chemotherapy only groups. Fifty-two patients were finally matched in the palliative chemotherapy only group.

### 4.3. Data Collection

Initial patients’ disease states before palliative chemotherapy were classified according to the biological categories of classification, based on the classification of stage IV gastric cancer by Yoshida et al. [12]. In brief, patients without macroscopic peritoneal seedings were further classified into category 1 (potentially resectable metastasis, i.e., single liver metastasis, few para-aortic lymph node: 16a2, b1) and category 2 (marginally resectable metastases, i.e., liver metastatic lesion >1, liver tumor size >5 cm, liver lesion close to the hepatic vein or the portal vein, distant lung metastasis, Virchow’s node metastasis, or para-aortic lymph nodes: 16a1, b2). Patients with macroscopic peritoneal seeding were classified into category 3 (incurable, no involvement of contiguous organs), and category 4 (incurable metastases, invasion into other organs). CT scans were conducted to measure the extent of the disease and evaluate the response after chemotherapy. Tumor responses were divided into CR, PR, SD, NE, and progressive disease (PD), according to the Response Evaluation Criteria in Solid Tumors version 1.1. All patients and tumor information were retrieved from the electronic medical record.

### 4.4. Expression of Cancer Dormancy Marker

**Figure 3:**
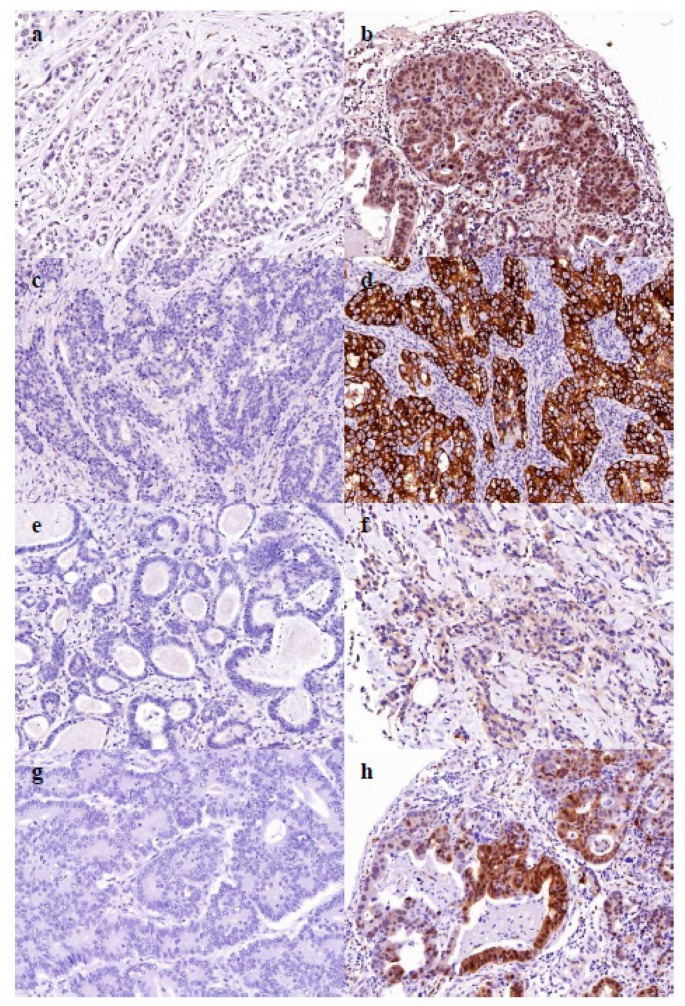
Tissue microarray (TMA) core of the cancer dormancy markers. (**a**) Negative expression of the NR2F1. (**b**) Positive expression of the NR2F1. (**c**) Negative expression of the NANOG. (**d**) Positive expression of the NANOG. (**e**) Negative expression of the MIG6. (**f**) Positive expression of the MIG6. (**g**) Negative expression of the PERK. (**h**) Positive expression of the PERK.

TMA was used for the analysis of the expression of cancer dormancy markers, including NR2F1, NANOG, MIG6, and PERK. TMAs were generated as described below. Tissue samples from endoscopic biopsy were fixed in 10% buffered formalin for 24–48 h, and then embedded in paraffin. The representative cores (2 mm in diameter) were isolated from the individual paraffin blocks and arranged in new tissue array blocks using a trephine apparatus (Superbiochips Laboratories, Seoul, Korea). Included cases had tumors occupying more than 10% of the core area. The TMA blocks contained up to 60 cores. The 4 μm sections from TMA blocks were stained with the following primary antibodies—rabbit monoclonal anti-NR2F1 (Abcam, Cambridge, MA, USA); rabbit monoclonal anti-NANOG (Abcam, Cambridge, MA, USA); rabbit polyclonal anti-MIG6 (Sigma-Aldrich, St. Louis, MO, USA); and rabbit monoclonal anti-PERK (Cell Signaling Technology, Danvers, MA, USA). Immunostaining was performed using the BenchMark XT platform (Ventana Medical Systems, Tucson, AZ, USA) according to the manufacturer’s instructions. The intensity of expression was interpreted as 0, 1+, 2+, and 3+. Intensity of 2+ or 3+ in at least 10% tumor cells was defined as positive expression; that of 0 or 1+ was defined as negative expression (Figure 3).

### 4.5. Statistical Analysis

Patient characteristics were compared in the matched cohorts via chi-square tests. Survival curves were compared using the Kaplan–Meier method by log-rank test. DFS was calculated from the time of conversion surgery to the first evidence of recurrence or death from any cause. We defined PFS as the time elapsed between the initiation of first-line palliative chemotherapy and disease progression or death from any cause. OS was measured from the initiation of first-line palliative chemotherapy to death from any cause or follow-up loss. A two-sided *p*-value of less than 0.05 was considered statistically significant. All statistical analyses were performed using the R software version 3.5.0.

This study was conducted in accordance with the ethical standards of the Declaration of Helsinki and the national and international guidelines. This study was approved by the institutional review board at SNUBH (B-1708/417-306 and B-1402/240-004).

## 5. Conclusions

In conclusion, patients with initially metastatic gastric cancer might benefit from conversion surgery and reach durable survival if the R0 resection can be achieved after chemotherapy. Although further studies are warranted for validation, the expression of cancer dormancy markers, i.e., NR2F1, might be predictive in achieving better postoperative survival outcome in patients undergoing conversion surgery.

## Figures and Tables

**Table 1:** Demographics and baseline characteristics of patients.

Variable	Conversion Surgery (*n* = 26) No. (%)	Chemotherapy Only		*p*-Value ^a^	*p*-Value ^b^
		Before Propensity Score Matching (*n* = 114) No. (%)	After Propensity Score Matching (*n* = 52) No. (%)		
Age (Median, Range)	58 (39–78)	61 (52–70)	57 (52–68)	0.855	1.000
<70 years	20 (76.9%)	83 (72.8%)	40 (76.9%)		
≥70 years	6 (23.1%)	31 (27.2%)	12 (23.1%)		
Sex				0.829	0.514
Male	18 (69.2%)	84 (73.7%)	41 (78.8%)		
Female	8 (30.8%)	30 (26.3%)	11 (21.2%)		
Metastatic site				0.355	0.935
Category 1–2	16 (61.5%)	56 (49.1%)	30 (57.7%)		
Category 3–4	10 (38.5%)	58 (50.9%)	22 (42.3%)		
1st line palliative chemotherapy ^c^				0.011	0.180
S1/capecitabine + cisplatin/oxaliplatin	14 (53.8%)	67 (58.8%)	31 (59.6%)		
FOLFOX	5 (19.2%)	36 (31.6%)	15 (28.8%)		
Herceptin + capecitabine + cisplatin	7 (26.9%)	6 (5.3%)	4 (7.7%)		
5-fluorouracil + cisplatin	0 (0.0%)	4 (3.5%)	1 (1.9%)		
Docetaxel + 5-fluorouracil + cisplatin	0 (0.0%)	1 (0.9%)	1 (1.9%)		
Best tumor response				0.298	1.000
CR, PR	17 (65.4%)	59 (51.8%)	34 (65.4%)		
SD, NE	9 (34.6%)	55 (48.2%)	18 (34.6%)		

^a^ Between conversion surgery (*n* = 26) and chemotherapy only group (*n* = 114), ^b^ Between conversion surgery (*n* = 26) and propensity score matched chemotherapy only group (*n* = 52). ^c^ Two of 26 patients in conversion surgery group and 8 of 114 patients in chemotherapy only group were under clinical trial and received combination therapy with an additional investigational agent including cetuximab, axitinib, and sunitinib. FOLFOX: oxaliplatin, 5-fluorouracil, leucovorin; CR, complete response; PR, partial response; SD, stable disease; NE, not evaluable for response.

**Table 2:** Clinicopathological characteristics of tumor in conversion surgery patients.

Variable	N (%)
Initial biological disease category before palliative chemotherapy	
Category 1	5 (19.2)
Category 2	11 (42.3)
Category 3	4 (15.4)
Category 4	6 (23.1)
Best tumor response before conversion surgery	
Complete response	2 (7.7)
Partial response	15 (57.7)
Stable disease	3 (11.5)
Not evaluable	6 (23.1)
Type of resection	
Subtotal gastrectomy	11(42.3)
Total gastrectomy	8 (30.8)
Extended total gastrectomy	7 (26.9)
R0 resection	
R0	22 (84.6)
R2	4 (15.4)
Lymphatic invasion	
Not identified	5 (19.2)
Present	21 (80.8)
Vascular invasion	
Not identified	15 (57.7)
Present	11 (42.3)
Perineural invasion	
Not identified	9 (34.6)
Present	17 (65.4)
Lauren classification	
Intestinal	13 (50.0)
Diffuse	10 (38.5)
Indeterminate	1 (3.8)
Others	2 (7.7)
Histologic differentiation	
Tubular adenocarcionma	16 (61.5)
Poorly cohesive carcinoma	5 (16.2)
Papillary adenocarcinoma	3 (11.5)
No tumor	2 (7.7)
TNM ^a^	
ypT	
0	1 (3.8)
1	2 (7.7)
2	3 (11.5)
3	12 (46.2)
4	7 (26.9)
ypN	
0	8 (30.8)
1	2 (7.7)
2	6 (23.1)
3	10 (38.5)
Postoperative stage	
0	2 (7.7)
I	2 (7.7)
II	3 (11.5)
III	10 (38.5)
IV	9 (34.6)

^a^ AJCC, American Joint Committee on Cancer 8th edition. One case was diagnosed as poorly differentiated adenocarcinoma at initial preoperative endoscopic biopsy, but the immunostaining of the resected tumor revealed a large cell neuroendocrine carcinoma component.

**Table 3:** The characteristics and clinical course of patients who showed long-term survival (> 3 years) among patients with conversion surgery.

Case No.	Age (Years)	Sex	Initial Metastatic Sites	Initial Biological Category Before Palliative Chemotherapy	Initial Chemotherapy	Chemotherapy Duration Before Conversion Surgery (Months)	Chemotherapy Response	Operation	Curativity	TNM Stage	Maintenance Chemotherapy	Recur	Survival Status	Overall Survival (Months)
1	64	M	Liver	2	FOLFOX	4.0	PR	TG + D2 + intraoperative radiofrequency ablation (scar change)	R0	pT2N1	Yes	No	alive	142.2
2	65	F	Liver and pancreas invasion	2	FOLFOX	3.8	CR	STG + D2	R0	pT0N0	No	No	alive	91.2
3	45	M	Peritoneal seeding, paraaortic LN	4	XELOX	4.1	PR	extended TG + D3 dissection	R0	pT0N0	Yes	No	alive	66.5
4	46	F	Retroperitoneal LN	2	XP	3.1	PR	STG + D3	R0	pT1N2	No	Yes	alive	61.8
5	56	F	Portocaval LN	2	XP + Herceptin	3.8	SD	STG + D3	R0	pT3N3a	Yes	No	alive	56.2
6	47	F	Peritoneal seeding	3	XELOX	4.7	NE	TG + D2	R0	pT2N0	Yes	Yes	alive	50.9
7	43	M	Retroperitoneal LN	1	XP + Herceptin	4.9	PR	STG + D3	R0	pT1N0	Yes	No	alive	47.6
8	51	M	Peritoneal seeding, Retroperitoneal LN	4	TS1 + Cisplatin	10.1	SD	TG + D3	R0	pT4aN3bM1(LN #16b1, #14)	Yes	Yes	expired	46.5
9	73	M	Peritoneal seeding, Colon and pancreas invasion	4	FOLFOX	3.2	CR	STG + D2	R0	pT2N0M1 (LN #13)	No	No	alive	46.0
10	65	M	Retroperitoneal LN	2	XP	11.1	PR	STG + D3	R2	T3N2M1(residual lesion at cardia)	Yes	Yes	expired	43.6
11	57	M	Peritoneal seeding	3	XELOX	13.9	SD	TG + D2	R0	pT3N2	No	Yes	alive	40.3
12	76	M	Pancreas body, and gallbladder invasion	2	XP + Herceptin	4.4	PR	STG + D2 + cholecystectomy + LN dissection (#8)	R0	pT3N2	Yes	No	alive	38.5
13	56	M	Retroperitoneal LN	2	TS1 + Cisplatin	5.0	NE	STG + D2 (no visual retroperitoneal LN)	R0	pT3N0	Yes	No	alive	37.1

LN, lymph nodes; FOLFOX folinic acid, fluorouracil, leucovorin, oxaliplatin; XELOX capecitabine and oxaliplatin; XP capecitabine and cisplatin; TS1, Tegafur/gimeracil/oteracil; CR, complete response; PR, partial response; SD, stable disease; NE, not evaluable for response; STG, subtotal gastrectomy; TG, total gastrectomy; TNM, tumor-node-metastasis.

## Supplementary Materials

The following are available online at https://www.mdpi.com/2072-6694/12/1/86/s1, Figure S1: DFS from conversion surgery with R0 resection according to cancer dormancy marker expression, Table S1: The characteristics and clinical course of patients who received noncurative resection with conversion surgery.

## References

[1] Jung K.W., Won Y.J., Kong H.J., Lee E.S. (2019). Prediction of Cancer Incidence and Mortality in Korea, 2019. Cancer Res. Treat..

[2] Bray F., Ferlay J., Soerjomataram I., Siegel R.L., Torre L.A., Jemal A. (2018). Global cancer statistics 2018: GLOBOCAN estimates of incidence and mortality worldwide for 36 cancers in 185 countries. CA.

[3] Guideline Committee of the Korean Gastric Cancer Association (KGCA), Development Working Group & Review Panel (2019). Korean Practice Guideline for Gastric Cancer 2018: An Evidence-based, Multi-disciplinary Approach. J. Gastric Cancer.

[4] Fujitani K., Yang H.K., Mizusawa J., Kim Y.W., Terashima M., Han S.U., Iwasaki Y., Hyung W.J., Takagane A., Park D.J. (2016). Gastrectomy plus chemotherapy versus chemotherapy alone for advanced gastric cancer with a single non-curable factor (REGATTA): A phase 3, randomised controlled trial. Lancet Oncol..

[5] Du R., Hu P., Liu Q., Zhang J. (2019). Conversion Surgery for Unresectable Advanced Gastric Cancer: A Systematic Review and Meta-Analysis. Cancer Investig..

[6] Einama T., Abe H., Shichi S., Matsui H., Kanazawa R., Shibuya K., Suzuki T., Matsuzawa F., Hashimoto T., Kohei N. (2017). Long-term survival and prognosis associated with conversion surgery in patients with metastatic gastric cancer. Mol. Clin. Oncol..

[7] Fukuchi M., Mochiki E., Ishiguro T., Kumagai Y., Ishibashi K., Ishida H. (2018). Prognostic Significance of Conversion Surgery Following First- or Second-line Chemotherapy for Unresectable Gastric Cancer. Anticancer Res..

[8] Fukuchi M., Mochiki E., Ishiguro T., Ogura T., Sobajima J., Kumagai Y., Ishibashi K., Ishida H. (2017). Efficacy of Conversion Surgery Following S-1 plus Cisplatin or Oxaliplatin Chemotherapy for Unresectable Gastric Cancer. Anticancer Res..

[9] Kim K.H., Lee K.W., Baek S.K., Chang H.J., Kim Y.J., Park D.J., Kim J.H., Kim H.H., Lee J.S. (2011). Survival benefit of gastrectomy ± metastasectomy in patients with metastatic gastric cancer receiving chemotherapy. Gastric Cancer.

[10] Mieno H., Yamashita K., Hosoda K., Moriya H., Higuchi K., Azuma M., Komori S., Yoshida T., Tanabe S., Koizumi W. (2017). Conversion surgery after combination chemotherapy of docetaxel, cisplatin and S-1 (DCS) for far-advanced gastric cancer. Surg. Today.

[11] Morgagni P., Solaini L., Framarini M., Vittimberga G., Gardini A., Tringali D., Valgiusti M., Monti M., Ercolani G. (2018). Conversion surgery for gastric cancer: A cohort study from a western center. Int. J. Surg..

[12] Yoshida K., Yamaguchi K., Okumura N., Tanahashi T., Kodera Y. (2016). Is conversion therapy possible in stage IV gastric cancer: The proposal of new biological categories of classification. Gastric Cancer.

[13] Paez D., Labonte M.J., Bohanes P., Zhang W., Benhanim L., Ning Y., Wakatsuki T., Loupakis F., Lenz H.J. (2012). Cancer dormancy: A model of early dissemination and late cancer recurrence. Clin. Cancer Res..

[14] Katayama H., Kurokawa Y., Nakamura K., Ito H., Kanemitsu Y., Masuda N., Tsubosa Y., Satoh T., Yokomizo A., Fukuda H. (2016). Extended Clavien-Dindo classification of surgical complications: Japan Clinical Oncology Group postoperative complications criteria. Surg. Today.

[15] Cook J.A. (2009). The challenges faced in the design, conduct and analysis of surgical randomised controlled trials. Trials.

[16] Kim S.W. (2014). The result of conversion surgery in gastric cancer patients with peritoneal seeding. J. Gastric Cancer.

[17] Fukuchi M., Ishiguro T., Ogata K., Kimura A., Kumagai Y., Ishibashi K., Ishida H., Kuwano H., Mochiki E. (2015). Risk Factors for Recurrence After Curative Conversion Surgery for Unresectable Gastric Cancer. Anticancer Res..

[18] Fukuchi M., Ishiguro T., Ogata K., Suzuki O., Kumagai Y., Ishibashi K., Ishida H., Kuwano H., Mochiki E. (2015). Prognostic Role of Conversion Surgery for Unresectable Gastric Cancer. Ann. Surg. Oncol..

[19] Nakamura M., Ojima T., Nakamori M., Katsuda M., Tsuji T., Hayata K., Kato T., Yamaue H. (2019). Conversion Surgery for Gastric Cancer with Peritoneal Metastasis Based on the Diagnosis of Second-Look Staging Laparoscopy. J. Gastrointest. Surg..

[20] Beom S.H., Choi Y.Y., Baek S.E., Li S.X., Lim J.S., Son T., Kim H.I., Cheong J.H., Hyung W.J., Choi S.H. (2018). Multidisciplinary treatment for patients with stage IV gastric cancer: The role of conversion surgery following chemotherapy. BMC Cancer.

[21] Yuan S.Q., Nie R.C., Chen S., Chen X.J., Chen Y.M., Xu L.P., Yang L.F., Zhou Z.W., Peng J.S., Chen Y.B. (2017). Selective Gastric Cancer Patients with Peritoneal Seeding Benefit from Gastrectomy after Palliative Chemotherapy: A Propensity Score Matching Analysis. J. Cancer.

[22] Zurleni T., Gjoni E., Altomare M., Rausei S. (2018). Conversion surgery for gastric cancer patients: A review. World J. Gastrointest. Oncol..

[23] Cunningham D., Allum W.H., Stenning S.P., Thompson J.N., Van de Velde C.J., Nicolson M., Scarffe J.H., Lofts F.J., Falk S.J., Iveson T.J. (2006). Perioperative chemotherapy versus surgery alone for resectable gastroesophageal cancer. N. Engl. J. Med..

[24] Thompson V.C., Day T.K., Bianco-Miotto T., Selth L.A., Han G., Thomas M., Buchanan G., Scher H.I., Nelson C.C., Greenberg N.M. (2012). A gene signature identified using a mouse model of androgen receptor-dependent prostate cancer predicts biochemical relapse in human disease. Int. J. Cancer.

[25] Borgen E., Rypdal M.C., Sosa M.S., Renolen A., Schlichting E., Lonning P.E., Synnestvedt M., Aguirre-Ghiso J.A., Naume B. (2018). NR2F1 stratifies dormant disseminated tumor cells in breast cancer patients. Breast Cancer Res..

[26] Miki Y., Tokunaga M., Tanizawa Y., Bando E., Kawamura T., Terashima M. (2015). Staging Laparoscopy for Patients with cM0, Type 4, and Large Type 3 Gastric Cancer. World J. Surg..

[27] Yasufuku I., Nunobe S., Ida S., Kumagai K., Ohashi M., Hiki N., Sano T. (2019). Conversion therapy for peritoneal lavage cytology-positive type 4 and large type 3 gastric cancer patients selected as candidates for R0 resection by diagnostic staging laparoscopy. Gastric Cancer.

