# Supplementary material for: Conversion Surgery in Metastatic Gastric Cancer and Cancer Dormancy as a Prognostic Biomarker
Source: Cancers (Basel). 2019 Dec 30;12(1):86. doi: 10.3390/cancers12010086 (PMC7016667; doi:10.3390/cancers12010086)
Supplement: Supplementary file 1 [file cancers-12-00086-s001.pdf]

Supplementary Materials: Conversion Surgery in Metastatic Gastric Cancer and Cancer Dormancy as a Prognostic Biomarker

Hun Jee Choe, Jin Won Kim, Song-Hee Han, Ju Hyun Lee, Sang-Hoon Ahn, Do Joong Park, Ji-Won Kim, Yu Jung Kim, Hye Seung Lee, Jee Hyun Kim, Hyung-Ho Kim and Keun-Wook Lee

Table S1. The characteristics and clinical course of patients who received noncurative resection with conversion surgery.

Case No.	Age (Years)	Sex	Initial Metastatic Sites	Initial Biological Category before Palliative Chemotherapy	Initial Chemotherapy	Chemotherapy Duration before Conversion Surgery (Months)	chemotherapy Response	Operation	Curativity	TNM Stage	Maintenance Chemotherapy	Recur	Survival Status	Overall Survival (Months)
1	65	M	Retroperitoneal LN	2	XP	11.1	PR	DG with uncut R-en-Y anastomosis	R2	pT3N2M1	Yes	Yes	expired	43.6
2	58	M	Peritoneal seeding, paraaortic LN	3	XP + Herceptin	5.1	NE	TG	R2	pT4N3M1	Yes	Yes	expired	27.4
3	39	F	paraaortic LN	2	Sunitinib + XELOX	4.9	PR	extended TG + D3 dissection	R2	pT4N3M1	Yes	Yes	expired	12.2
4	75	M	Celiac axis, pancreas, Peritoneal seeding	4	TS1 + Oxaliplatin	8.8	NE	TG + right hemicolectomy, peritonectomy	R2	pT3N2M1	Yes	Yes	expired	14.7

LN, lymph nodes; XELOX capecitabine and oxaliplatin; XP capecitabine and cisplatin; TS1, Tegafur/gimeracil/oteracil; PR, partial response; NE, not evaluable for response; TG, total gastrectomy; DG, distal gastrectimy; TNM, tumor-node-metastasis.

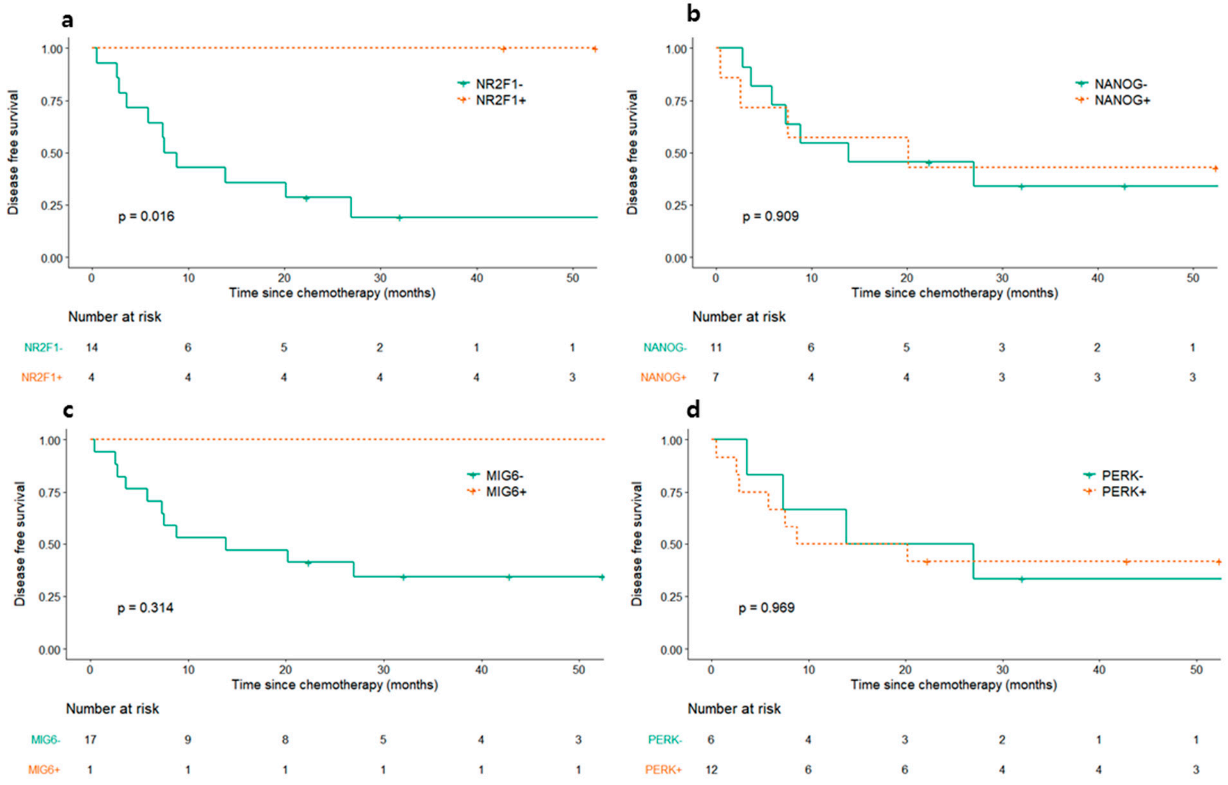

Figure S1. DFS from conversion surgery with R0 resection according to cancer dormancy marker expression.

© 2020 by the authors. Licensee MDPI, Basel, Switzerland. This article is an open access article distributed under the terms and conditions of the Creative Commons Attribution (CC BY) license (http://creativecommons.org/licenses/by/4.0/).
